# Effects of a 48-Day Home Quarantine during the Covid-19 Pandemic on the First Outdoor Running Session among Recreational Runners in Spain

**DOI:** 10.3390/ijerph18052730

**Published:** 2021-03-08

**Authors:** Manuel Mosqueira-Ourens, José M. Sánchez-Sáez, Aitor Pérez-Morcillo, Laura Ramos-Petersen, Andrés López-Del-Amo, José L. Tuimil, Adrián Varela-Sanz

**Affiliations:** 1 Departament of Podiatry, Faculty of Health Science, Campus de Los Jerónimos, Universidad Católica San Antonio de Murcia, Guadalupe, 30107 Murcia, Spain; mjmosqueira@ucam.edu (M.M.-O.); jmssaez@ucam.edu (J.M.S.-S.); lrpetersen@ucam.edu (L.R.-P.); alopez@ucam.edu (A.L.-D.-A.); 2 ABIDOR, Research Group “Avances en Biomecánica Deportiva y Ortopodología”, Campus de Los Jerónimos, Universidad Católica San Antonio de Murcia, Guadalupe, 30107 Murcia, Spain; 3 Department of Physical Education and Sport, University of La Coruña, Bastiagueiro, 15179 Oleiros, Spain; jose.luis.tuimil@udc.es (J.L.T.); adrian.varela.sanz@udc.es (A.V.-S.)

**Keywords:** COVID-19, confinement, running, physical activity

## Abstract

COVID-19-induced quarantine may lead to deleterious effects on health status as well as to impaired performance and increased injury risk when re-starting training after lockdown. We investigated the physical activity (PA) habits of recreational runners in Spain during a 48-day home quarantine during the COVID-19 pandemic and the characteristics of the first outdoor running session after confinement. A cross-sectional study, including a self-reported running questionnaire completed after the first outdoor running session after quarantine, was performed. Three hundred recreational runners (74% males; 60% 18–40 years old; most typical running experience >3 years, 10–30 km weekly running distance distributed in 3–4 sessions) were considered for analysis. Advanced runners ran, at least, 4 days/week and participated in running events. They performed significantly longer and more non-supervised weekly training sessions during confinement (*p* < 0.01 for both) than novice and amateur runners. Most runners performed their first outdoor running session on asphalt (65.3%) and ran 5 to 10 km (61%) at a pace above 5 min/km (60%), reporting no pain before (77%), during (64%), and 24 h after (76%) the session. Advanced runners performed a significantly longer running session, at a higher pace, and covered a greater distance (*p* < 0.01 for all) than novice and amateur runners, while enjoyment and motivation tended to be significantly higher when runners’ level increased (*p* < 0.05). Higher training levels prior to and during confinement may lower the collateral effects (e.g., detraining, injury risk) of home quarantine when runners return to previous PA levels.

## 1. Introduction

At the end of 2019, SARS-CoV-2 (severe acute respiratory syndrome coronavirus 2) infection, a novel virus emerged in the city of Wuhan, China, and expanded worldwide, causing Corona Virus Disease 2019 (COVID-19), an etiologically unknown pneumonia [1,2]. In March 2020, the World Health Organization (WHO) declared COVID-19’s global pandemic status. This pandemic is producing a devastating impact not only on public health but also on society’s lifestyle and the economy of the entire world [1,3].

In an attempt to slow the spread of COVID-19, and therefore reduce death rates, countries adopted different prevention measures as a function of the severity of their own situation, including social distancing, wearing of surgical masks, limitation of social interactions, school closures, cancellation of major international sports events and championships (e.g., Summer Olympics, UEFA European Football Championship), travel restrictions, and home quarantine [2,4,5]. In fact, most countries of the world imposed an obligatory home quarantine at some point, such as in Spain, where the government announced the lockdown of the country on 15 March 2020 [6]. During this period, citizens could only leave their homes to go grocery shopping, get medical care, or go to the pharmacy. In this regard, initiating a sudden quarantine state implies a radical change in the lifestyle of the population [7]. Recent scientific data suggest that isolation strategies like confinement have collateral effects, such as drastic reductions in daily physical activity (PA) levels and social interactions, as well as important increases in sedentary behavior while maintaining or even increasing daily energy intake (i.e., producing a positive energy balance) [3,4,8,9,10,11,12].

On 2 May 2020, after 48 days of home quarantine, the population living in Spain could finally leave their homes to exercise or simply go for a walk again [13]. In this sense, recreational endurance running has become one of the most practiced PAs not only in Spain but worldwide [14]. In fact, participation in running events in the last 10 years increased in the entire world almost 58% [15]. The success of this massive practice could be related to its simplicity and accessibility, being a low-cost option and a useful tool for maintaining or increasing healthy fitness [16,17]. However, the COVID-19 pandemic significantly reduced not only the number of endurance and ultra-endurance running events worldwide but also the number of runners who finished races [18]. Considering the actual situation, it was suggested that during prolonged home isolation-induced quarantine athletes are likely exposed to some level of detraining due to a reduction or cessation in their training routine with substantial declines in daily PA [5,19]. Mujika and Padilla analyzed, in an excellent two-part review, the effects of detraining on training-induced adaptations in the short- (i.e., <4 weeks of insufficient training stimulus) and the long-term (i.e., >4 weeks of insufficient training stimulus) [20,21]. Short-term detraining leads to impaired endurance performance due to important declines in maximal oxygen uptake (VO_2max_) and lactate threshold in highly trained athletes, while these changes are more moderate in individuals with lower-level PA [20]. On the other hand, insufficient training stimuli for more than 4 weeks (i.e., long-term) produce greater declines in VO_2max_ levels and lactate threshold with associated impaired endurance performance [21]. Similarly, training-induced neuromuscular adaptations (e.g., fiber cross-sectional area, fiber type, muscle architecture, tendon properties, and neural drive to the spinal-motor pool) can be negatively affected by training cessation in the short- (e.g., neural alterations) and long-term (e.g., morphological changes) [19]. In this regard, it was previously suggested that insufficient training stimuli may increase injury risk when returning to previous daily PA levels, in part due to an alteration of the tissue-specific mechanical properties as a consequence of decreasing the amount and the specificity of training loads during quarantine [5,17]. Recent scientific data have demonstrated that COVID-19-induced quarantine produces more detrimental effects on performance than traditional off-season in professional soccer players [22]. Similarly, 5- and 20-min cycling performance in professional cyclists significantly declined up to 19% after lockdown [23], while only two weeks of detraining seems to be enough for reducing cardiopulmonary function in well-trained endurance athletes [24].

Considering that professional and non-professional athletes of different sport disciplines had to face an unprecedented and relatively long-term reduction or cessation in their training routines due to COVID-19 pandemic-induced confinement [19], to the best of our knowledge this topic was not yet explored among recreational runners of different levels. Therefore, the main objective of our investigation was to study the PA habits of recreational runners living in Spain during a 48-day home quarantine during the COVID-19 pandemic and the characteristics of the first outdoor running session after confinement. We hypothesized that the higher the runner’s level, the better the response in the first outdoor running session after confinement.

## 2. Materials and Methods

### 2.1. Study Design

Our investigation was a cross-sectional study including the COVID-19 Running Questionnaire, a self-reported questionnaire delivered via Google Forms to study the PA habits of habitual and non-habitual recreational runners living in Spain during a 48-day home quarantine during the COVID-19 pandemic and the characteristics of the first outdoor running session after confinement. The inclusion criteria were to be over 18 years, to run at least 1–2 times per week, and to perform an outdoor running training session after the 48-day quarantine period in Spain. The exclusion criteria were incomplete or incongruent as well as duplicate responses. Our survey included questions about participants’ training characteristics before the 48-day home quarantine, physical activity habits at home during the 48-day quarantine, characteristics of the first outdoor running session after the 48-day quarantine, and effects on some self-perceived variables (i.e., physical and affective variables) before, during, and after the first outdoor running session after the 48-day quarantine. All participants reported their own level based on a runners’ level classification developed by the research group. For this investigation, runners’ level was described as follows: Novice runners: “I am starting to run, and I do not have a training program”; Amateur runners: “I run 1–3 times per week without a training program, and I participate in some running events”; Advanced runners: “I have a scheduled training program, and I run at least 4 days per week. I also habitually participate in running events”.

### 2.2. Data Collection

The COVID-19 Running Questionnaire was specifically designed for this investigation by the research group and announced through social media in different universities, athletic clubs, and fitness centers across the country and delivered the evening of 2 May 2020 (i.e., the first day after the 48-day quarantine period when people living in Spain could leave home to exercise outdoors), via the Google Forms platform, guaranteeing anonymity and confidentiality of participants at all times. Data were collected over a 1-week period until 10 May 2020.

Ethical review and approval were waived for this study due to the COVID-19 pandemic situation at the time of the study in Spain (please see the Institutional Review Board Statement for further details). This investigation committed to all criteria included in the Declaration of Helsinki for Human Research and was supported by the research group Advances in Sports Biomechanics and Orthopedics (ABIDOR) from the Catholic University of Murcia (UCAM) in Spain. Participation was completely voluntary and anonymous, and participants were informed about the objectives of the study at the beginning of the questionnaire. Participants were also informed that data collected contained no personal data and would be strictly used only for research and scientific divulgation purposes. If a participant decided to answer and send the questionnaire, personal informed consent was assumed.

### 2.3. Participants

After the 1-week period, data from 359 habitual and non-habitual recreational runners living in Spain were collected. Responses were filtered by two researchers, and cases were removed when necessary (i.e., incomplete or incongruent responses, duplicate responses, not meeting the inclusion criteria). After data filtration, 300 participants were included for data analyses.

### 2.4. Statistical Analyses

All data were analyzed with the statistical package IBM SPSS Statistics 20 (SPSS Inc. Chicago, IL, USA). Categorical variables are presented for the entire sample and male and female subgroups and are summarized using counts and percentages. For all categorical variables, a chi-square test or a contingency coefficient test was performed as required to compare subgroups (i.e., males vs females, runners’ self-reported levels). To determine the association power between categorical variables and its direction a Kendall Tau-b test was used as required. Statistical significance was set at *p* < 0.05.

## 3. Results

### 3.1. Training Characteristics before the 48-Day Home Quarantine

Training characteristics of recreational runners in Spain are presented in Table 1. The final study sample was composed of 74% males and 26% females, all of them were ≥18 years old. Regarding running experience above 3 years, there were significantly more males than females within this group (C = 0.25, *p* = 0.001). Further, a significant trend was found for men to run more than 40 km/week and women between 21 and 30 km/week (x^2^ (4, *n* = 300) = 13.24, *p* = 0.01). Women were more likely to be supervised by a sport science professional or by a coach previous to quarantine than men (x^2^ (1, *n* = 300) = 4.56, *p* = 0.033). There were no more significant differences between males and females for the rest of the comparisons (*p* > 0.05).

Regarding the runners’ self-reported level, advanced runners presented significantly more running experience (C = 0.44, *p* < 0.001; Tau-b = 0.38, *p* < 0.001), performed more running sessions per week (C = 0.53, *p* < 0.001; Tau-b = 0.53, *p* < 0.001), and covered a greater weekly distance (x^2^ (8, *n* = 300) = 168.95, *p* < 0.001; (Tau-b = 0.58, *p* < 0.001)) than novice and amateur runners. Interestingly, advanced runners were more likely to be supervised by a sport science professional or by a coach previous to quarantine than novice and amateur runners (x^2^ (2, *n* = 300) = 27.54, *p* < 0.001; (Tau-b = −0.29, *p* < 0.001)). There were no more significant differences between runners’ levels for the rest of comparisons (*p* > 0.05).

### 3.2. Physical Activity Habits at Home during the 48-Day Home Quarantine

Table 2 shows the physical activity habits at home of the sample during the 48-day home quarantine. Males who were not supervised by a sport science professional or by a coach performed significantly more resistance exercises than females (C = 0.30, *p* = 0.002). There were no more significant differences between males and females for the rest of comparisons (*p* > 0.05).

Regarding the runners’ self-reported level, advanced runners were more supervised by a sport science professional or by a coach during the 48-day home quarantine than novice and amateur runners (x^2^ (2, *n* = 300) = 19.11, *p* < 0.001; (Tau-b = −0.24, *p* < 0.001)). Further, advanced runners significantly performed longer (x^2^ (6, *n* = 193) =18.29, *p* = 0.006; (Tau-b = 0.26, *p* < 0.001)) and more (x^2^ (6, *n* = 193) = 19.5, *p* = 0.003; (Tau-b = 0.27, *p* < 0.001)) non-supervised weekly training sessions than novice and amateur runners. There were no more significant differences between runners’ levels for the rest of comparisons (*p* > 0.05).

### 3.3. Characteristics of the First Outdoor Running Session

Characteristics of the first outdoor running session after the 48-day home quarantine are presented in Table 3. Female runners who ran at a pace above 5 min/km were significantly more than male runners (C = 0.27, *p* < 0.001). Moreover, female runners alternated running and brisk walking significantly more than males (x^2^ (1, *n* = 300) =14.47, *p* < 0.001)). There were no more significant differences between males and females for the rest of comparisons (*p* > 0.05).

Regarding the runners’ self-reported level, a significant trend was found for novice and amateur runners to alternate brisk walking and running during the first outdoor running session (x^2^ (2, *n* = 300) = 28.95, *p* < 0.001; (Tau-b = 0.3, *p* < 0.001)). Further, advanced runners performed a significantly longer running session (x^2^ (6, *n* = 300) = 21.16, *p* = 0.002; (Tau-b = 0.21, *p* < 0.001)) at a higher pace (C = 0.43, *p* < 0.001; Tau-b = −0.18, *p* = 0.002) and covered a greater distance (C = 0.37, *p* < 0.001; Tau-b = 0.32, *p* < 0.001) than novice and amateur runners. There were no more significant differences between runners’ levels for the rest of comparisons (*p* > 0.05).

### 3.4. Effects on Self-Perceived Variables before, during, and after the First Outdoor Running Session

Table 4 shows the effects on self-perceived variables before, during, and after the first outdoor running session after the 48-day home quarantine. In relation to the affective variables experienced during the running session, female runners experienced significantly higher motivation levels (C = 0.16, *p* = 0.04) and better feelings than males (x^2^ (4, *n* = 300) = 10.91, *p* = 0.03). There were no more significant differences between males and females for the rest of comparisons (*p* > 0.05).

On the other hand, enjoyment (C = 0.24, *p* = 0.007; Tau-b = 0.21, *p* < 0.001) and motivation (C = 0.22, *p* = 0.021; Tau-b = 0.19, *p* < 0.001) during the first outdoor running session tended to be significantly higher when the runners’ level increased. There were no more significant differences between runners’ levels for the rest of comparisons (*p* > 0.05).

## 4. Discussion

To the best of our knowledge, our investigation is the first study to investigate the effects of a home quarantine on an outdoor running session among recreational runners. Considering that PA levels of recreational runners during COVID-19-induced confinement might be higher than those of the physically inactive/sedentary population, our results confirmed our hypothesis. After a 48-day home quarantine, the majority of our runners ran between 5 and 10 km, at a pace above 5 min/km, and on asphalt and reported no pain before, during, and 24 h after the running session. Covered running distance, running training session time, and running pace were higher when runners’ level increased (i.e., better response). This fact could be related to the pre-pandemic training characteristics of our sample, suggesting that a higher training level prior to and during confinement may lower the collateral effects (e.g., detraining, injury risk) of home quarantine when returning to previous daily PA levels.

Since the WHO declared in March 2020 a global pandemic, the majority of the countries in the world adopted different prevention measures and restrictions during the so-called “first wave” in an attempt to slow the spread of COVID-19. These actions resulted in a great threat to human society in terms of economy and lifestyle, but they were necessary to protect public health [3]. Some of the actual restrictions such as curfew, selective home quarantine, and closure of sport facilities are still making it challenging to remain physically active or to achieve optimal training stimuli levels. Scientific evidence has recently suggested that COVID-19-induced home confinement significantly decreased the time expended in PA at all intensity levels, whereas daily sedentary behavior increased [25,26,27,28,29]. In this regard, it was consistently demonstrated that even acute short periods of reduced levels of PA may have detrimental health effects. For instance, 14 days of step reduction from a high (~10,000 steps/day) to a low level (~1500 steps/day) leads to a cardiorespiratory fitness loss up to ~7% mL/kg/min, which can result in a reduction of life expectancy, and muscle atrophy in the lower extremities even in young, healthy adults [9].

Pre-pandemic data derived from the Eurobarometer showed that nearly half of Europeans never exercise or play sport (in Spain, non-exercisers constitute 46% of the population), and this proportion showed a gradual increase since 2009 [30]. However, recreational endurance running has become one of the most practiced PAs, not only in Spain but worldwide [14], increasing the participation in running events almost 58% in the last 10 years [15]. Nevertheless, recent scientific data demonstrated that during the COVID-19 pandemic the number of endurance and ultra-endurance events significantly decreased, as well as did the number of runners who finished races [18], suggesting that professional and non-professional athletes of different sport disciplines are facing an unprecedented and relatively long-term reduction or cessation in their training routines [19].

Our results suggest that the great majority of our participants regularly performed some PA during confinement. From 300 participants, only 7 reported not exercising at home during this period. In fact, the majority of our runners performed 3–6 home-based training sessions/week with durations between 31 and 60 min/training session. The fact that almost all participants regularly performed PA could be due to their pre-pandemic PA levels (i.e., training characteristics), since ~79% of runners presented a running experience above 3 years, with almost half of them running between 10 and 30 km/week with a training frequency of 3–4 days/week. Moreover, males presented significantly more running experience than females and tended to run more than 40 km/week. Our results are in accordance with previous scientific evidence [14] that showed that recreational runners living in Spain run 1–3 times/week, with men accumulating significantly more years of running experience and training more hours/week than women. However, one can assume that training loads (i.e., weekly covered distance and exercise volume and intensity) were reduced during confinement; unfortunately, we did not collect this information. The most preferred non-supervised PA performed by our sample at home during confinement were resistance exercises (~38%), with males performing significantly more of this activity than females, while walking was the second most practiced PA (~30%). These results are partially in accordance with López-Martínez et al., who reported that people living in Spain who exercised at home without supervision during quarantine preferably chose intensity-related exercises and resistance exercises. However, these authors reported a walking prevalence of only 11% [31].

On the other hand, as counteracting strategies to fight reduced levels of training during the COVID-19 pandemic, some elite sports clubs provided their athletes with home-based training programs and/or organized video conferences for online training sessions [5]. If there are already difficulties for professional sports clubs in implementing appropriate training programs during confinement, it would be reasonable to assume that those difficulties are exponentially greater for non-professional/recreational athletes, since they do not have the same resources, support, and access to professional knowledge as those who are professionals. With this consideration, it was suggested that during home confinement athletes are likely exposed to some level of detraining due to a reduction or cessation in their training routine [5,19]. The short- (i.e., <4 weeks) and the long-term (>4 weeks) effects of detraining result in impaired endurance performance due to marked declines in VO_2max_ and lactate threshold [20,21], as well as important losses in neuromuscular training-induced adaptations [19]. This situation could predispose athletes to be more susceptible to injury through an alteration of the tissue-specific mechanical properties [5], the knee being the most common body location for lower extremity injuries in recreational runners [17]. Previous scientific evidence in professional sports (i.e., National Football League lockout) reported a higher rate of Achilles tendon injuries after an uncommon 4-month off-season, after which athletes returned to training camp and in the beginning of preseason [32]. Since our investigation was a cross-sectional study, we cannot evaluate the real effects of a confinement period on running-related injury risk. However, the fact that the majority of our participants did not experience physical pain could be linked to their pre-confinement training characteristics and to the PA performed at home during confinement. Assuming that the absence of sports-related injuries is utopian, appropriate running volume and intensity, especially in novice runners, and the use of adequate running shoes, might contribute to reducing the likelihood of injury among recreational runners [17]. In this sense, the time required to attain pre-detraining cardiorespiratory and neuromuscular levels is determined by different factors, such as time of training stimuli cessation or reduction, amount of individual detraining-induced effects, individual fitness level, and sport-specific requirements, and may considerably vary between individuals [19].

Previous studies have demonstrated that the main motivational reasons among recreational runners are to have fun, to obtain health benefits, and to achieve personal goals [14,33]. Self-perceived affective variables analyses during the first outdoor running session in our study showed that enjoyment and motivation tended to be significantly higher when the runner’s level increased. Further, female runners experienced significantly higher motivation levels and better feelings than males. In this regard, it has been recently suggested that competitive runners are more motivated than their recreational counterparts for performance [33]; nevertheless, motivation of recreational runners is high in task (i.e., personal goal and mastery achievement) and low in ego (i.e., competition and external achievement/social recognition) orientation [14,33].

## 5. Conclusions

Considering that PA levels of recreational runners during COVID-19-induced confinement might be higher than those of the physically inactive/sedentary population, our results suggest that a higher training level prior to and during a 48-day home quarantine may lower the collateral effects (e.g., detraining, injury risk) of home confinement when returning to previous daily PA levels.

Limitations and future research lines

One of the limitations of our investigation is the lack of a standardized and validated questionnaire. For future research, it would be valuable to evaluate the PA levels of recreational runners during confinement in terms of volume and intensity distribution in order to get a better understanding of the effects on training-induced adaptations, performance outcomes, and injury risk.

## Figures and Tables

**Table 1:** Training characteristics of recreational runners in Spain before the 48-day home quarantine.

			All Participants (*n* = 300)		Males (*n* = 222)		Females (*n* = 78)	
			*n*	%	*n*	%	*n*	%
	Age (years)	18–30	89	29.7	63	28.3	26	33.3
		31–40	92	30.7	71	32	21	26.9
		41–50	80	26.7	60	27	20	25.7
		51–60	35	11.6	25	11.3	10	12.8
		>60	4	1.3	3	1.4	1	1.3
**Training Characteristics**	Running experience	<6 months	8	2.7	6	2.7	2	2.6
		6 months–1 year	15	5	6	2.7	9	11.5
		1–2 years	19	6.3	10	4.5	9	11.5
		2–3 years	18	6	10	4.5	8	10.3
		>3 years	236	78.7	186	83.8	50	64.1
		N.A.	4	1.3	4	1.8	0	0
	Weekly running sessions (days)	1–2	83	27.6	57	25.6	26	33.3
		3–4	165	55	127	57.2	38	48.7
		5–6	47	15.7	35	15.8	12	15.4
		Every day	5	1.7	3	1.4	2	2.6
	Weekly running distance (km)	<10	56	18.7	36	16.2	20	25.6
		10–20	73	24.3	53	23.8	20	25.6
		21–30	74	24.7	49	22.1	25	32.1
		31–40	42	14	37	16.7	5	6.4
		>40	55	18.3	47	21.2	8	10.3
	Self-reported level	Novice	40	13.3	27	12.2	13	16.7
		Amateur	137	45.7	98	44.1	39	50
		Advanced	123	41	97	43.7	26	33.3
	Pronation pattern	Neutral	113	37.7	84	37.9	29	37.2
		Supination	36	12	28	12.6	8	10.3
		Overpronation	76	25.3	62	27.9	14	17.9
		I do not know	75	25	48	21.6	27	34.6
	Supervised by a coach before the quarantine	Yes	181	60.3	126	56.8	55	70.5
		No	119	39.7	96	43.2	23	29.5

N.A.: not answered.

**Table 2:** Physical activity habits at home during the 48-day home quarantine of recreational runners in Spain.

			All Participants (*n* = 300)		Males (*n* = 222)		Females (*n* = 78)	
			*n*	%	*n*	%	*n*	%
**Physical Activity Habits at Home during the 48-Day Home Quarantine**	Supervised by a coach during the quarantine	Yes	100	33.3	70	31.5	30	38.5
		No	200	66.7	152	68.5	48	61.5
	**Participants supervised by a coach during the quarantine (*n* = 100)**							
	Supervised training sessions	Yes	100	100	70	100	30	100
		No	0	0	0	0	0	0
	Supervised weekly training sessions	1–2	18	18	13	18.6	5	16.6
		3–4	28	28	20	28.6	8	26.7
		5–6	35	35	26	37.1	9	30
		Every day	19	19	11	15.7	8	26.7
	Supervised average training session duration (min)	<30	2	2	1	1.4	1	3.3
		31–45	21	21	15	21.4	6	20
		46–60	63	63	48	68.6	15	50
		>60	14	14	6	8.6	8	26.7
	**Participants not supervised by a coach during the quarantine (*n* = 200)**							
	Non-supervised training sessions	Yes	193	96.5	146	96.1	47	97.9
		No	7	3.5	6	3.9	1	2.1
	Non-supervised physical activities	Walking	57	29.5	42	28.7	15	31.9
		Yoga	19	9.8	9	6.2	10	21.3
		Pilates	7	3.6	3	2.1	4	8.5
		Zumba	5	2.6	4	2.7	1	2.1
		Res. Exerc.	74	38.3	65	44.5	9	19.2
		Others	31	16.1	23	15.8	8	17
	Non-supervised weekly training sessions	1–2	31	16.1	23	15.7	8	17
		3–4	64	33.1	49	33.6	15	31.9
		5–6	60	31.1	47	32.2	13	27.7
		Every day	38	19.7	27	18.5	11	23.4
	Non-supervised average training session duration (min)	<30	29	15	26	17.8	3	6.4
		31–45	70	36.3	51	34.9	19	40.4
		46–60	62	32.1	46	31.5	16	34
		>60	32	16.6	23	15.8	9	19.2

Res. Exerc.: resistance exercises; Walking was performed at home or around the house.

**Table 3:** Characteristics of the first outdoor running session after the 48-day home quarantine of recreational runners in Spain.

			All Participants (*n* = 300)		Males (*n* = 222)		Females (*n* = 78)	
			*n*	%	*n*	%	*n*	%
**First Outdoor Running Session Characteristics after the 48-Day Home Quarantine**	Use of specific running shoes	Yes	279	93	205	92.3	74	94.9
		No	21	7	17	7.7	4	5.1
	Running shoes recommendation	Podiatrist	77	25.7	57	25.6	20	25.6
		Coach	58	19.3	46	20.7	12	15.4
		Physiotherapist	15	5	13	5.9	2	2.6
		Vendor	66	22	38	17.1	28	35.9
		Others	63	21	51	23	12	15.4
		N.A.	21	7	17	7.7	4	5.1
	Use of running accessories	Orth. Insoles	72	24	55	24.8	17	21.8
		Comp. Stockings	6	2	6	2.7	0	0
		Running socks	63	21	46	20.7	17	21.8
		None of them	159	53	115	51.8	44	56.4
	Warm-up including stretching	Yes	186	62	130	58.6	56	71.8
		No	114	38	92	41.4	22	28.2
	Running surface	Asphalt	196	65.3	145	65.3	51	65.4
		Ground/Mountain	88	29.3	66	29.7	22	28.2
		Grass	6	2	2	0.9	4	5.1
		Sand	5	1.7	4	1.8	1	1.3
		Others	5	1.7	5	2.3	0	0
	Running session duration (min)	<30	54	18	40	18	14	17.9
		31–45	100	33.3	77	34.7	23	29.5
		46–60	105	35	74	33.3	31	39.7
		>60	41	13.7	31	14	10	12.8
	Running pace (min:s)	<3:30	4	1.3	1	0.4	3	3.8
		3:31–4:00	4	1.3	1	0.4	3	3.8
		4:01–4:30	18	6	16	7.2	2	2.6
		4:31–5:00	68	22.7	61	27.5	7	9
		>5:00	180	60	126	56.8	54	69.2
		I do not know	26	8.7	17	7.7	9	11.5
	Running distance (km)	<5	63	21	42	18.9	21	26.9
		5–10	183	61	134	60.3	49	62.8
		>10	46	15.3	39	17.6	7	9
		I do not know	8	2.7	7	3.2	1	1.3
	Alternation of running and brisk walking	Yes	156	52	101	45.5	55	70.5
		No	144	48	121	54.5	23	29.5
	Cool-down including stretching	Yes	217	72.3	155	69.8	62	79.5
		No	83	27.7	67	30.2	16	20.5

N.A.: not answered; Orth. Insoles: orthopedic insoles; Comp. Stockings: compression stockings.

**Table 4:** Effects on self-perceived variables before, during and after the first outdoor running session after the 48-day home quarantine of recreational runners in Spain.

				All Participants (*n* = 300)		Males (*n* = 222)		Females (*n* = 78)	
				*n*	%	*n*	%	*n*	%
**First Outdoor Running Session Self-Perceived Variables**	**Physical variables before, during and after the running session**	Pain before the running session	Foot sole	18	6	16	7.2	2	2.6
			Achilles tendon	13	4.3	9	4	4	5.1
			Knee	20	6.7	16	7.2	4	5.1
			Hip	6	2	3	1.4	3	3.9
			Back	11	3.7	6	2.7	5	6.4
			Other	0	0	0	0	0	0
			I had no pain	232	77.3	172	77.5	60	76.9
		Pain during the running session	Foot sole	21	7	14	6.3	7	9
			Achilles tendon	16	5.3	12	5.4	4	5.1
			Knee	36	12	29	13	7	9
			Hip	14	4.6	8	3.6	6	7.7
			Back	11	3.7	7	3.2	4	5.1
			Other	11	3.7	7	3.2	4	5.1
			I had no pain	191	63.7	145	65.3	46	59
		Session interrupted due to pain	Yes	13	4.3	8	3.6	5	6.4
			No	96	32	69	31.1	27	34.6
			I had no pain	191	63.7	145	65.3	46	59
		Pain ≥24 h after the running session	Foot sole	16	5.3	12	5.4	4	5.1
			Achilles tendon	15	5	12	5.4	3	3.8
			Knee	21	7	17	7.6	4	5.1
			Hip	5	1.7	3	1.4	2	2.6
			Back	11	3.6	6	2.7	5	6.4
			Other	5	1.7	5	2.3	0	0
			I had no pain	227	75.7	167	75.2	60	76.9
		Skin injuries after the running session	Blisters	30	10	23	10.3	7	9
			Toenails injuries/pain	9	3	7	3.2	2	2.6
			Heel rash/wounds	4	1.3	2	0.9	2	2.6
			I had no injuries	257	85.7	190	85.6	67	85.8
	**Affective variables during the running session**	Enjoyment	Nothing	0	0	0	0	0	0
			Little	6	2	5	2.2	1	1.3
			Normal	26	8.7	23	10.4	3	3.8
			Some	114	38	87	39.2	27	34.6
			Much	154	51.3	107	48.2	47	60.3
		Motivation	Nothing	0	0	0	0	0	0
			Little	4	1.3	3	1.4	1	1.3
			Normal	28	9.3	21	9.5	7	9
			Some	135	45	110	49.5	25	32
			Much	133	44.4	88	39.6	45	57.7
		Perceived effort	Nothing	14	4.7	8	3.6	6	7.7
			Little	77	25.6	57	25.7	20	25.6
			Normal	132	44	102	45.9	30	38.5
			Some	72	24	52	23.4	20	25.6
			Much	5	1.7	3	1.4	2	2.6
		Feelings	Very bad	2	0.7	2	0.9	0	0
			Bad	25	8.3	18	8.1	7	9
			Normal	63	21	54	24.3	9	11.5
			Good	149	49.7	111	50	38	48.7
			Very good	61	20.3	37	16.7	24	30.8

## Data Availability

The data presented in this study are available on request from the corresponding author. The data are not publicly available due to privacy.
